# Prevalence of intestinal and haemoprotozoan parasites of small ruminants in Tamil Nadu, India

**DOI:** 10.14202/vetworld.2015.1205-1209

**Published:** 2015-10-17

**Authors:** R. Velusamy, N. Rani, G. Ponnudurai, P. Anbarasi

**Affiliations:** Department of Veterinary Parasitology, Veterinary College and Research Institute, Tamil Nadu Veterinary and Animal Sciences University, Namakkal - 637 002, Tamil Nadu, India

**Keywords:** helminths, haemoprotozoan parasites, prevalence, sheep, goats

## Abstract

**Aim:**

The aim of the present study is to assess the prevalence of intestinal and haemoprotozoan parasites of small ruminants (Sheep and Goats) in North Western part of Tamil Nadu, India.

**Materials and Methods:**

A total of 630 faecal samples (251-sheep, 379-goats) and 554 blood smears (242-sheep, 312-goats) were examined, for the presence of eggs of intestinal and haemoprotozoan parasites, respectively. The samples were received from the Veterinary college hospital and Veterinary dispensaries in North Western part of Tamil Nadu. Faecal samples were processed by sedimentation technique and examined under low power objective (×10), and blood smears were stained using Giemsa’s technique and examined under oil immersion (×100).

**Result:**

The analysis of data on the prevalence of intestinal and haemoprotozoan parasites of sheep and goats in North Western part of Tamil Nadu for the period from 2004 to 2013, showed an overall prevalence of intestinal parasites was found to be 67% and 35% in sheep and goats, respectively, whereas only 11% of sheep and 3% of goats had the haemoprotozoan parasitic infection. Highly, significant difference (p<0.01) in the prevalence of intestinal (χ^2^=65), and hemoprotozoan (χ^2^=15.4) parasitism was observed between sheep and goats. Intestinal parasites such as strongyles, *Trichuris, Moniezia*, amphistome, and coccidia were identified in which the highest prevalence was observed with coccidia, followed by strongyles, *Monezia, Trichuris*, and least with amphistome in both the sheep and goats. The haemoprotozoan parasites recorded were *Theileria* and *Anaplasma* species, of which, *Anaplasma* spp. being the highest and *Theileria* spp. the least prevalent in both the sheep and goats. The seasonal prevalence of intestinal parasites showed highest in rainy season, followed by moderate in winter and least with summer in both the sheep and goats, whereas the haemoprotozoan parasites recorded were the highest in summer followed by winter and least with rainy season.

**Conclusion:**

The present study suggests that North Western part of Tamil Nadu is highly endemic for intestinal parasites such as coccidia and strongyles and haemoprotozoans such as *Anaplasma* and *Theileria* species in small ruminants.

## Introduction

Gastrointestinal (GI) parasitism is one of the major health problems affecting productivity of small ruminants worldwide [1]. GI parasitic infection in sheep and goats are of much economic importance because, small ruminants rearing has become a major source of income especially for the poor marginal farmers in rural areas of India [2,3].

Recurring losses in productivity due to widely prevalent parasitic infection is important and common recurrent problem for small ruminant’s production in most parts of the world [4]. Vast studies on the prevalence of GI parasites have been documented from different parts of India [5-10] and a few numbers in Tamil Nadu [11]. In addition to GI parasitic infection, small ruminants are also highly susceptible to haemoprotozoan parasites [12]. The tropical environment is the major reason for the development of these parasitic diseases [13].

A proper understanding of the epidemiology of parasitic diseases is a prerequisite for the rational design for the effective preventive and control measures against the dreadful parasitic diseases. Although most of the studies have been carried out with respect to epidemiology of blood and gastrointestinal parasitism in large animals, there is no much study on small ruminants in North Western part of Tamil Nadu, hence, the present study was undertaken to assess the parasitic infection in small ruminants.

## Materials and Methods

### Ethical approval

Samples were collected from clinical cases coming to Veterinary hospital at Veterinary College and Research Institute, Namakkal. So, this particular study does not require ethical approval.

### Study area

Faecal samples and blood smears were received from the Veterinary college hospital and Veterinary dispensaries in and around Namakkal area, which is located in North Western part of Tamil Nadu. The geographical location of the study lies between 11.00 and 11.360 North latitude and 77.280 and 78.300 East longitude and witnessed a temperature range of 35-38°C with maximum of 42°C, relative humidity of 57-55% and rainfall about 1-4. The season in this area can be broadly classified into hot and dry summer from March to June, rainy (monsoon) season from July to October and the winter (mild) season from November to February.

### Study period

The data recorded in the specimen register of Department of Veterinary Parasitology were compiled and analyzed for a period of 10 years from January 2004 to December 2013.

### Sample size

A total of 630 faecal samples (251-sheep, 379-goats) and 554 blood smears (242-sheep, 312-goats) were examined, for the presence of eggs of intestinal parasites and haemoprotozoan parasites, respectively.

### Faecal sample examination

Faecal samples submitted to Department of Veterinary Parasitology were processed by sedimentation technique and examined under low power objective (×10). The ova of intestinal parasites were identified based on their morphological features [14].

### Blood smear examination

Thin blood smears received from the Veterinary College Hospital and Veterinary dispensaries were fixed in methanol (5 min) and stained with Giemsa’s stain (30 min) [15] and examined under oil immersion (100 X magnifications), for the presence of blood parasites. The parasites were identified based on their characteristic morphology [16].

### Statistical analysis

Data were statistically analyzed using Pearson Chi-squared test at p<0.01 regarded as statistically significant [17], and Microsoft Excel was used for presentation of the results.

## Results

**Figure-1:**
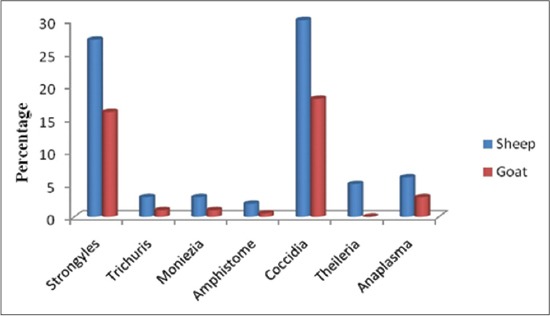
Species wise prevalence of intestinal and haemoprotozoan parasites in sheep and goats.

**Figure-2:**
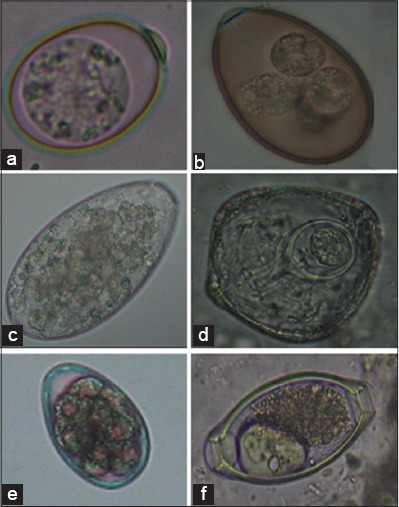
Ova of different species of intestinal parasites in sheep and goats. (a) Unsporulated oocyst of Eimeira sp - goat (40X), (b) Sporulated oocyst Eimeira sp - goat (40X), (c) Egg of Amphistome- sheet (40X), (D) Egg of Moniezia sp - Sheep (40X), (E) Embryonated egg of Strongyle sp- Goat (40X), (F) Egg of Trichuris sp- Sheep (40X)

The analysis of data on the prevalence of intestinal and haemoprotozoan parasites of sheep and goats in North Western part of Tamil Nadu for the period from 2004 to 2013, showed an overall prevalence of intestinal parasites was found to be 67% and 35% in sheep and goats, respectively, whereas only 11% of sheep and 3% of goats had the haemoprotozoan parasitic infection (Table-1). Highly, significant difference (p<0.01) in the prevalence of intestinal (χ^2^=65) and haemoprotozoan (χ^2^=15.4) parasitism was observed between sheep and goats. Intestinal parasites such as strongyles, *Trichuris, Moniezia*, amphistome, and coccidia were identified in which the highest prevalence was observed with coccidia, followed by strongyles, *Monezia, Trichuris* and least with amphistome in both the sheep and goats (Figures-1 and 2). The haemoprotozoan parasites recorded were *Theileria* and *Anaplasma* species, of which, *Anaplasma* spp. being the highest and *Theileria* spp. the least prevalent in both the sheep and goats. The seasonal prevalence of intestinal parasites showed highest in rainy season, followed by moderate in winter and least with summer in both the sheep and goats whereas the haemoprotozoan parasites recorded were highest in summer, followed by winter and least with rainy season (Table-2). Highly, significant difference (p<0.01) in the prevalence of intestinal and haemoprotozoan parasitism was also observed among different seasons in sheep and goats.

**Table-1:** Overall prevalence of intestinal and haemoprotozoan parasites in sheep and goats.

Animals	Total number of faecal samples examined	Intestinal parasites			Total number of blood smears examined	Blood parasites		
	
		Number of positive	Percentage	χ^2^ value		Number of positive	Percentage	χ^2^ value
Sheep	251	169	67	65.0**	242	27	11	15.4**
Goats	379	131	35		312	9	3	

** Value with similar superscript in a column is highly significant (p>0.01)

**Table-2:** Seasonal prevalence of intestinal and haemoprotozoan parasites in sheep and goats.

Season	Intestinal parasites						Blood parasites					
	
	Sheep			Goats			Sheep			Goats		
			
	Number of faecal samples examined	Number of positive (%)	χ^2^ value	Number of faecal samples examined	Number of positive (%)	χ^2^ value	Number of blood smears examined	Number of positive (%)	χ^2^ value	Number of blood smears examined	Number of positive (%)	χ^2^ value
Summer	56	29 (51)	14.9**	79	9 (11)	25.6**	80	22 (28)	32.5**	72	6 (8)	8.40*
Monsoon	97	79 (81)		146	65 (45)		66	1 (2)		116	1 (1)	
Winter	98	68 (69)		154	57 (37)		96	4 (4)		124	3 (2.4)	

** Value with similar superscript in a column is highly significant (p>0.01)

## Discussion

Among the intestinal parasites observed in this study, coccidian infections were predominant in both the sheep and goats. This result is in conformity with the findings from Namakkal reported the higher incidence of *Eimeria* spp. in 34.61% of sheep [18] and 26.57% of goats in Greater Kamrup district of Assam [19] and similar findings are also reported from Nigeria, the high prevalence of coccidia was observed in both the lambs and kids [20]. The high prevalence of coccidiosis in small ruminants obtained in this study could be as a result of the management system operated by most small ruminants’ owners especially during the rainy season when animals are confined to avoid damage to crops. Consequently, such animals are overcrowded in the pens, which are not properly cleaned regularly. These factors with the high humidity of the rainy season predispose them to high parasitic infections. Next to coccidia, strongyle infection was observed high in both the sheep and goats in this study. The observed high prevalence rate of intestinal nematodes agrees with the findings of earlier investigators [20-22]. It was reported that the prevailing climatic conditions especially rainfall and temperature favor the development and survival of parasitic nematode eggs of infective stages [23]. The least infection of amphistome in sheep and goats may be due to the presence of fewer water bodies in the study, which limited the accessibility of infection through snails.

An effort that was made to know the influence of seasonal variation on the prevalence of helminths infection was found to be significantly high during monsoon, followed by moderate in winter and least in summer in both the sheep and goats. The present investigation is in conformity with the report from Maiduguri, Nigeria [24] a high prevalence of *Haemonchus* and *Trichostrongylus* species were encountered during rainy season and attained peak counts at the same time in both goats (June) and sheep (August). In other study from Tamil Nadu recorded a significantly higher helminthic infection during Northeast monsoon followed by Southwest monsoon, then winter and least infection during the summer season [25]. There was a definite seasonal influence in faecal egg counts of the sheep and goats and this corresponded with the pattern of rainfall. Environmental conditions are usually favorable for the development, survival and translocation of pre-parasitic stages during the rainy season. Therefore, there is a gradual build-up of adult worm populations in grazing animals so that higher prevalence of helminths recorded during the rainy season. Thereafter, sustained during winter and declined during dry season. In contrary to the present finding, higher percentage of parasitic infection was also observed in goats of the subtropical area of J and K during summer followed by winter, spring and lowest in autumn [26]. The difference could be due to the seasonal dynamics influencing ecological and environmental conditions of the study area.

The haemoprotozoan parasites recorded in this study showed highest in summer, followed by winter and least with rainy season. This study is in agreement with our previous studies in cattle [27] reported that prevalence of theileriosis was significantly higher during summer, followed by moderate in monsoon and less in winter season. The species of haemoprotozoan parasites reported in this study were similarly observed by Takeet *et al*. [28] in sheep that *Anaplasma ovis* is the most prevalent haemoprotozoan parasite in both sheep and goats [29]. A relatively high incidence of the haemoprotozoan parasite could be attributed to the favorable environmental conditions for the survival and transmission dynamics of the arthropod vectors. Considerable seasonal variation with respect to the occurrence of the haemoprotozoan disease may be due to changes in macroclimate that is essential for breeding of ticks.

## Conclusion

The present study suggests that North Western part of Tamil Nadu is highly endemic for coccidia and strongyles and *Anaplasma* in small ruminants. The result of this study clearly shows that most of the small ruminants kept in the area of study are infected with blood and intestinal parasites.

### Recommendation

The outcome of the present study would help to forecast disease outbreak not only in this region but also applicable to other parts of the country where similar type of climatic condition prevails. Prevention and control programs against these parasites of sheep and goats in endemic areas will improve the production potentials of these animals and the economic well-being of the marginal farmers. There is a need for further investigations using molecular techniques for the accurate identification of the carrier status of haemoprtozoan parasites in small ruminants.

## Authors’ Contributions

RV, NR, GP, and PA: Blood smear examination and identification of parasites. RV and GP:Preparation of manuscript and analysis of data. All authors read and approved the final manuscript.
